# Spinal cord size as promising biomarker of disability outcomes after hematopoietic stem cell transplantation in multiple sclerosis

**DOI:** 10.1016/j.msard.2024.105745

**Published:** 2024-06-26

**Authors:** Alice Mariottini, Emily H. Stack, Govind Nair, Chiara Nozzoli, Tianxia Wu, Leonardo Marchi, Riccardo Boncompagni, Anna Maria Repice, Enrico Fainardi, Francesca Di Pasquale, Edoardo Carlesi, Riccardo Saccardi, Steven Jacobson, Luca Massacesi

**Affiliations:** a Department of Neurofarba, University of Florence, Florence, Italy; b Department of Neurology 2 and Tuscan Region Multiple Sclerosis Referral Centre, Careggi University Hospital, Florence, Italy; c National Institute of Neurological Disorders and Stroke, NIH, Bethesda, MD, 20892 USA; d Cell Therapy and Transfusion Medicine Unit, Careggi University Hospital, Florence, Italy; e Neuroradiology Unit, Department of Experimental and Clinical Biomedical Sciences “Mario Serio”, University of Florence, Florence, Italy; f Neuroradiology Unit, Department of Radiology, Careggi University Hospital, Florence, Italy; g Diagnostic Imaging Unit, Department of Experimental and Clinical Biomedical Sciences, University of Florence, Florence, Italy

**Keywords:** Multiple sclerosis, Transplant, Atrophy, Spinal cord, Disability, Magnetic resonance imaging, hematopoietic stem cell transplantation

## Abstract

**Background:**

Biomarkers predictive of disability outcomes in individual multiple sclerosis (MS) patients undergoing autologous haematopoietic stem cell transplantation (AHSCT) are currently lacking. As correlations between spinal cord atrophy and clinical disability in MS were previously described, in this study spinal cord size was investigated in MS patients treated with AHSCT, exploring whether baseline spinal cord volume may predict disability progression after AHSCT.

**Methods:**

relapsing-remitting (RR-) and secondary-progressive (SP-) MS patients treated with AHSCT (BEAM/ATG regimen) at a single academic centre in Florence, who performed at least two standardized brain magnetic resonance imaging (MRIs) scans (acquired between one-year pre-AHSCT to 5 years after AHSCT) were included. Cervical spinal cord atrophy was estimated as upper cervical spinal cord cross-sectional area (SCCSA). Brain volume loss (BVL) was analysed at the same timepoints.

**Results:**

Eleven (8 RR-; 3 SP-) MS patients were included. Over a median follow-up of 66 (range 37 - 100) months, no relapses nor brain MRI activity were observed; disability progressed in 2 cases (both SP-MS). Baseline SCCSA was associated with EDSS change between pre- and one-year post-AHSCT. Compared to patients who stabilized, patients who progressed after AHSCT tended to have lower SCCSA at C4 level at baseline and year 1 after AHSCT. Longitudinal changes in SCCSA or BVL did not correlate with EDSS change.

**Conclusions:**

Baseline pre-AHSCT SCCSA, but not its longitudinal changes nor BVL, predicted EDSS change within the two years following AHSCT. SCCSA may represent a biomarker of treatment response and a promising screening tool for assessing patient eligibility for high-impact treatments such as AHSCT.

## 1. Introduction

Multiple sclerosis (MS) is a chronic inflammatory demyelinating and neurodegenerative autoimmune disease of the central nervous system (CNS) that may lead to cognitive and physical disability (Thompson et al., 2018). Despite the availability of high-efficacy disease-modifying therapies (DMTs), breakthrough disease activity may be observed in some cases. Such patients may be eligible for treatment with autologous haematopoietic stem cell transplantation (AHSCT), which is currently endorsed as a standard of care for relapsing MS refractory to conventional DMTs by the European and the American Bone and Marrow Transplantation Societies (Cohen et al., 2019; Sharrack et al., 2019). AHSCT is a multistep procedure inducing a durable abrogation of new focal inflammatory activity in MS, mediated by radical immunosuppression and subsequent immunoreconstitution. The latter is characterized by renewal of the T cell receptor repertoire (Muraro et al., 2005) and extensive modifications in immune cell networks towards a tolerogenic environment (Cencioni et al., 2021). As most chemotherapy drugs administered during AHSCT cross the blood-brain barrier (BBB) (Mariottini et al., 2020), it could be speculated that AHSCT may act also on chronic inflammation compartmentalized beyond a repaired BBB. However, experimental evidence supporting this hypothesis is currently lacking, partially due to the absence of validated biomarkers of compartmentalized inflammation in MS (Tommasin et al., 2019). AHSCT induces a persistent suppression of relapses and new focal inflammatory activity at magnetic resonance imaging (MRI) in the vast majority of the patients (Burt et al., 2022; Atkins et al., 2016), but its effect on disability accrual is heterogeneous across studies (Samijn et al., 2006; Tolf et al., 2019), although rates of progression-free survival (P-FS) are generally higher in relapsing-remitting (RR-) compared to progressive MS (Burt et al., 2022; PA Muraro et al., 2017). AHSCT is indeed highly effective in suppressing disability progression in RR-MS with short disease duration and low disability; on the other hand, progression irrespective of new focal inflammatory events (PIRA) may be observed in patients treated in the progressive phase of the disease, and in RR-MS who show moderate to severe disability at baseline (Atkins et al., 2016; Mariottini et al., 2022). Known predictors of response to AHSCT (i.e. young age, short disease duration, low EDSS, RR-MS course, and high inflammation) allow the identification of the “ideal candidate” for this procedure (PA Muraro et al., 2017). Whereas maximal benefit or detriment from AHSCT can be expected in the “ideal candidate” or its opposite, respectively, the prediction of individual response to AHSCT for patients sitting in the grey zone in between these two is challenging. In this area of uncertainty of outcomes, a biomarker that could predict Expanded Disability Status Scale (EDSS) (Kurtzke, 1983) progression would therefore improve the selection of patients eligible for high-impact procedures such as AHSCT, avoiding exposure to potentially life-threatening complications in those cases who would not likely gain any benefits from the procedure.

As disability measured with the EDSS is highly dependent on impairment in ambulation, spinal cord involvement plays a crucial role in determining EDSS score in moderately disabled patients (Hidalgo de la Cruz et al., 2022). The correlation between spinal cord atrophy and clinical disability was confirmed by a meta-analysis including 94 studies (Casserly et al., 2018), and spinal cord atrophy was suggested as the strongest MRI predictor of disability progression over follow-up (Tsagkas et al., 2018; Lukas et al., 2015).

In this paper, spinal cord atrophy was analysed for the first time as a surrogate marker and predictor of EDSS progression after AHSCT, as it might represent a screening tool to improve the selection of patients with uncertain clinical responses to this treatment.

## 2. Materials and methods

### Study design.

A retrospective multicentric study in MS patients treated with AHSCT aimed at investigating longitudinal changes in upper cervical spinal cord atrophy, also exploring whether pre-treatment upper cervical spinal cord cross-sectional area (SCCSA) could predict the occurrence of EDSS progression following AHSCT.

#### 2.1. Study participants

RR- or secondary-progressive (SP-) MS patients diagnosed according to the Poser and McDonald criteria (Poser et al., 1983; McDonald et al., 2001; Polman et al., 2005; Polman et al., 2011) treated with AHSCT in the period January 2011 – December 2019, and who had at least two brain MRI examinations with a standard protocol (including a 3D T1-weighted sequence at 1 mm isotropic resolution) and the same scanner (see section “MRI protocol” below) were included.

Transplants were performed at the Cell Therapy and Transfusion Medicine Unit of the Careggi University Hospital in Florence, Italy, in collaboration with the MS Referral Centre for the Tuscany region of the same hospital. Patients were treated with AHSCT for aggressive MS according to the inclusion/exclusion criteria of the transplant centre, as previously reported (Mariottini et al., 2022).

#### 2.2. Standard protocols approval, and patient consent

The protocol was approved by the local ethics committee (Tuscany region, Area Vasta Centro; approval number 14,399_bio); written informed consent was collected by each subject according to local regulations. The study has been performed in accordance with the ethical standards laid down in the 1964 Declaration of Helsinki and its later amendments, as well as in the observation of the specific national laws.

#### 2.3. AHSCT procedure

All the patients were treated with the same AHSCT protocol. Briefly, Peripheral Blood Haematopoietic Stem Cells (PBSC) were mobilised by the administration of cyclophosphamide (dose 4 g/m^2^ body surface area) followed by daily granulocyte colony-stimulating factor (G-CSF; 10 μg/kg per day) starting at day +5, until completion of the PBSC harvest by leukapheresis. The conditioning regimen used was BEAM/ATG, an intermediate intensity conditioning regimen according to the European Society for Blood and Marrow Transplantation (EBMT) classification (Sharrack et al., 2019), encompassing the following: BCNU (Carmustine) 300 mg/m^2^ on day −6, ARA-C (Cytosine-Arabinoside) 200 mg/m^2^/day and VP-16 (Etoposide) 200 mg/m^2^/day from day −5 to day −2, and Melphalan 140 mg/m^2^ on day −1; rabbit anti-thymocyte globulin (ATG, Thymoglobulin^™^, Sanofi) was added at a dose of 3.75 mg/kg/day on day +1 and +2 (total dose 7.5 mg/Kg).

Supportive therapies and infection prophylaxis were administered according to local protocols.

#### 2.4. Clinical examinations and outcomes

Standardized haematological and neurological evaluations (including EDSS assessment) were performed at baseline, at months 6 and 12 after AHSCT and then yearly.

EDSS increase was defined as an increase of at least 1.0 or 0.5 EDSS point if baseline EDSS was <5.5 or ≥5.5, respectively, confirmed at six months. Each event of EDSS increase was classified as either (i) single-step EDSS accrual (i.e. one single episode of EDSS increase, followed by EDSS stabilisation up to the last available follow-up); or (ii) EDSS progression (i.e. at least two episodes of EDSS increase associated with progression of disability between timepoints, corresponding to the maintenance of a progressive disease course), as previously reported (Mariottini et al., 2022).

Occurrence of relapses and new inflammatory activity at brain MRI (defined as the occurrence of new T2 lesions in a follow-up brain MRI compared to the re-baseline scan taken at month six after AHSCT, or of gadolinium-enhancing lesions at any time) was also recorded.

#### 2.5. MRI protocol

All the MRI studies were performed with the same 1.5 T scanner (MAGNETOM Symphony, Siemens Medical Systems, Erlangen, Germany) at the Neuroradiology Unit of the Careggi University Hospital in Florence, Italy. Baseline MRI was defined as the examination acquired within six months before AHSCT. Routine radiological follow-up included brain MRI carried out at months 6 and 12 after AHSCT, and yearly thereafter. Not all the MRI scans acquired at these pre-defined timepoints could be included in the analysis due to technical issues, mostly due to the lack of appropriate visualization of first cervical metamers. The MRI protocol included a volumetric FLAIR sequence, and at least one T1 sequence (T1 MPRAGE, T1 MTC and T13DSENSE TFE) acquired before and after gadolinium administration.

Baseline MRI was available in nine cases, eight of whom had at least one post-AHSCT examination. Two patients had post-AHSCT scans only. A pre-treatment scan (besides the baseline scan) taken 1 year before AHSCT was available in 2 cases. After AHSCT, the number of patients evaluated at month 6 and years 1, 2, 3, 4 and 5 was 5, 10, 6, 5, 4 and 2, respectively.

#### 2.6. Spinal cord cross-sectional area

3D T1-weighted images from the brain were analysed for SCCSA from C1 to C4 using software reported and evaluated in detail elsewhere (Mina et al., 2021; Azodi and Jacobson, 2020). Briefly, the matlab program reorients and automatically determines edges on the sagittal image, and the user manually selects the edges corresponding to the spinal cord. The software then calculates the SCCSA at each point using axial images reformatted perpendicular to the selected cord edge. Finally, the user identifies the region of the cord directly behind each vertebral disc, and the software outputs the average SCCSA corresponding to each vertebral segment. Since brain images were used, only the upper cervical spine, where each vertebral segment was clearly visualized, was used in determining the SCCSA.

#### 2.7. Brain volume change

Two-timepoint percentage brain volume change (PBVC) was estimated using the Structural Image Evaluation using Normalisation of Atrophy (SIENA) methodology (Smith et al., 2002; Smith et al., 2004); whole brain volume at a timepoint (normalized for subject head size) was calculated with SIENAX, FSL-suite. The annualized rate of brain volume loss (AR-BVL) was then calculated as follows: [(PBVC/100+1)^(365.25/days)−1]*100, where PBVC between two timepoints is measured using SIENA methodology, and “days” represents the number of days between the two scans, as previously reported (Kappos et al., 2016).

#### 2.8. Statistical methods

Baseline characteristics of patients are reported as median and range for quantitative variables and as frequency and percent for categorical variables. The cumulative proportion of survival free from single-step EDSS accrual (EDSS accrual-FS) or disability progression (P-FS) after AHSCT was estimated using Kaplan-Meier survival analysis, and the comparisons between the two MS forms were performed with a log-rank test. Spearman correlation coefficients were used to evaluate the relationship between spinal cord measures and clinical outcomes, such as between baseline SCCSA at C2 and the number of previous DMTs, or between SCCSA values and EDSS. Wilcoxon two-sample test was used to test the difference between the two groups and the t approximation p-values were reported. A two-tailed p-value <0.05 was considered significant. Considering the exploratory setting of this study, the analyses were not corrected for the multiplicity. A random coefficient model was applied to evaluate PBVC change over time. The follow-up year was treated as a continuous variable and a significance level of 0.1 was used for covariate selection. The normality assumption and outliers were checked using the Studentized residuals. A multiple logistic regression was performed to evaluate the effect of SCCSA measurements at baseline on the disease progression (the dependent variable). Age, disease duration, and number of previous DMTs were considered as covariates. Stepwise regression was used to select the covariate. None of them was selected at a significant level of 0.1. The simple logistic regression did not find any significant association between SCCSA and disease progression at follow-up.

The statistics software used were SAS 9.4 and SPSS version 25 (Windows); graphing with Origin Pro.

## 3. Results

### 3.1. Patient characteristics

Eleven (8 RR-, 3 SP-) MS patients were included (Table 1). The median clinical follow-up duration after AHSCT was 66 (range 37 – 100) months.

### 3.2. Relapses and disability following AHSCT

**Fig. 1.**
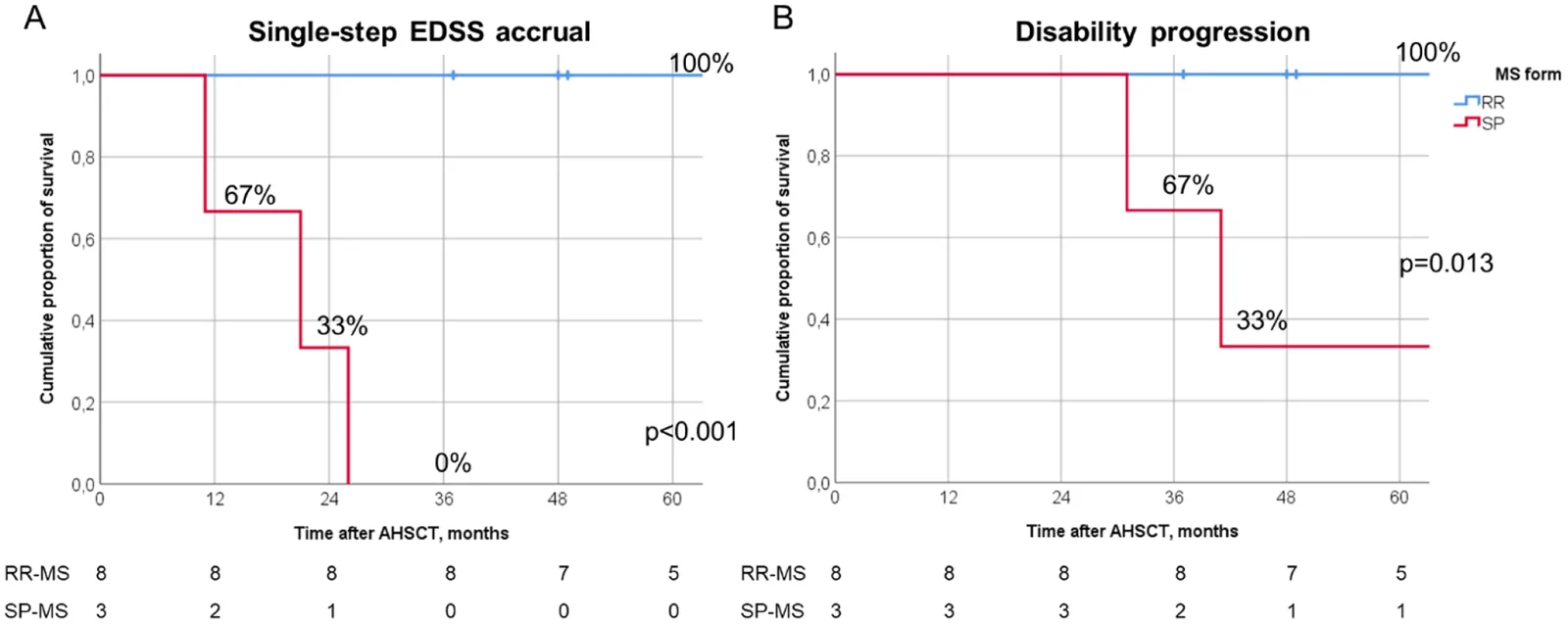
Cumulative proportion of survival free from single-step EDSS accrual (A) and EDSS progression (B) according to the MS subtype. The disability events occurred in patients with SP-MS only, whereas none of the RR-MS cases experienced EDSS increase. The number of patients at risk is reported below each chart.

No relapses nor new inflammatory activity at brain MRI were recorded up to the last follow-up after AHSCT. EDSS accrual-FS was 82 % at year 2 and 73 % at years 3 to 5 (data not shown). Considering all the patients, P-FS was 91 % at year 3 and 81 % at years 4–5: none of the RR-MS patients showed EDSS increase nor disability progression over the follow-up, whereas all the three SP-MS patients showed EDSS increase within 26 months from AHSCT (Fig. 1). Indeed, in two out of three SP-MS patients the progressive MS course was not substantially affected by AHSCT, as they experienced at least two distinct episodes of EDSS increase, being the second event observed at 41 and 31 months of follow-up, respectively. No gadolinium-enhancing lesions were detected in the spinal cord MRI, and the spinal cord lesion load was stable in the case who had a pre-AHSCT examination for comparison.

Patients who progressed (*n* = 2) tended to be an older age at AHSCT compared to those who did not (*n* = 9) (46.5 years vs 36; *p* = 0.0724), and the EDSS change both one year and two years following AHSCT was inversely correlated with the number of relapses in the 2 years before AHSCT (Spearman *r* = −0.80, *p* = 0.0054; and *r* = −0.93, *p* = 0.0081, respectively).

### 3.3. Spinal cord cross-sectional area

At baseline before AHSCT, median SCCSA was 61.3 (45.8 – 80.7) mm^2^ at C1, 54.2 (49.1 – 84.3) mm^2^ at C2, 53.3 (44.3 – 79.3) mm^2^ at C3, and 60.9 (36.6 – 85.6) mm^2^ at C4. SP-MS patients tended to have a lower SCCSA at C4 compared to RR-MS patients (36.8 mm^2^ vs 68.2 mm^2^; *p* = 0.071). Baseline area at C1 was inversely correlated with age at the time of transplant (*r* = −0.68, *p* = 0.042), and baseline area at C2 was inversely correlated with the number of previous DMTs (*r* = −0.68, *p* = 0.046).

When individual patients were evaluated longitudinally, there was no significant change in SCCSA from baseline at any region of the spinal cord (data not shown).

### 3.4. Correlation between spinal cord cross-sectional area and disability outcomes

Baseline SCCSA was strongly associated with EDSS change between pre- and one-year post- AHSCT. A larger increase in EDSS between these timepoints was observed in the patients with lower baseline SCCSA at C1 (Spearman *r* = −0.71, *p* = 0.0496), C3 (*r* = −0.73, *p* = 0.0390), and C4 (*r* = −0.89, *p* = 0.0073). Similarly, EDSS change at year two after AHSCT was associated with baseline SCCSA, but only at the C4 level (*r* = −0.90, *p* = 0.0374).

**Fig. 2.**
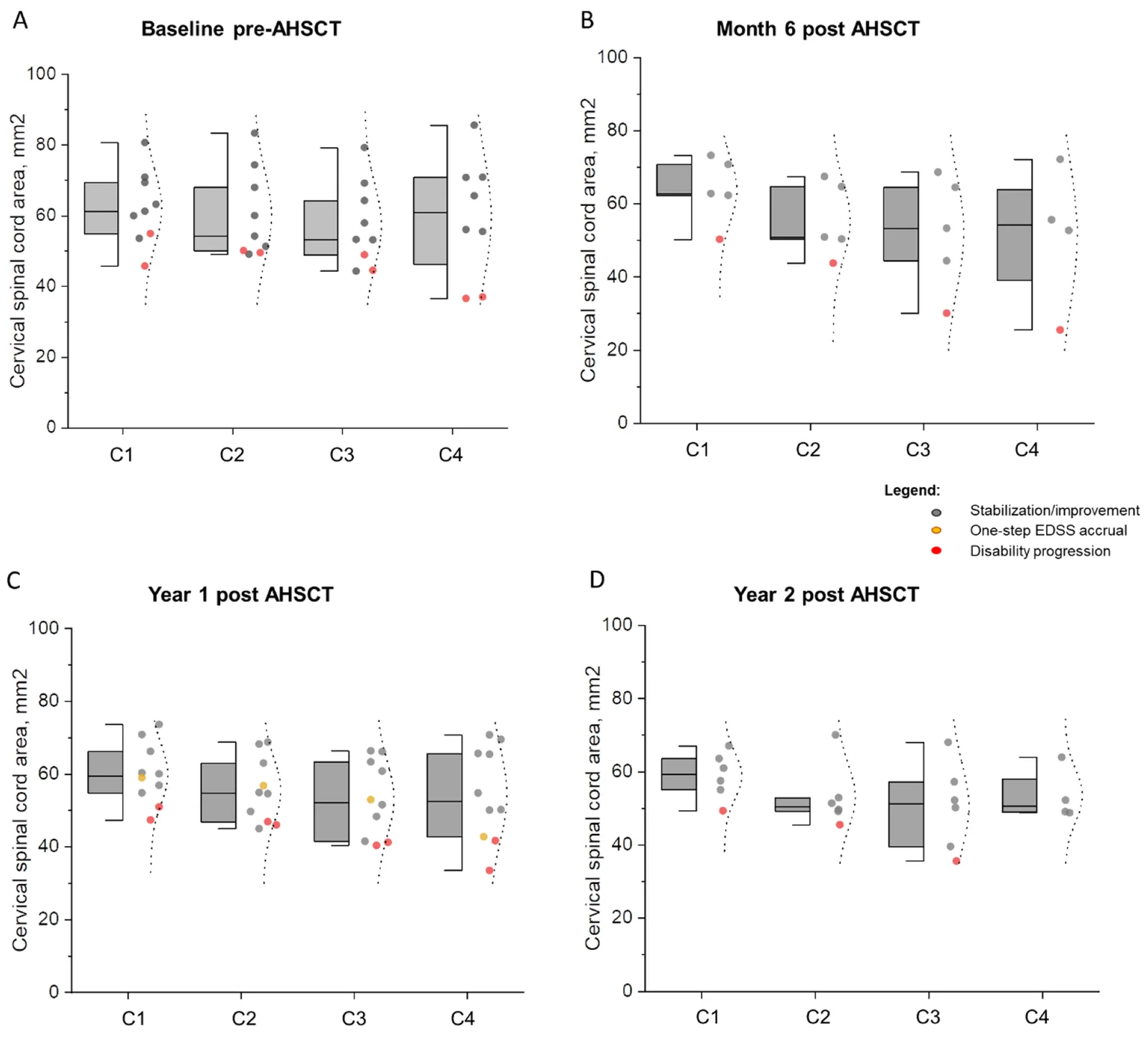
Median (range) and individual values of SCCSA measured at C1 to C4 before AHSCT (A) and up to year 2 after transplant (B-D). Patients who stabilized or improved are depicted in grey, whereas those who progressed or experienced a single-step EDSS increase are in red or yellow, respectively. Baseline SCCSA at C4 tended to be lower in cases who showed disability progression (red dots) compared to those who did not (*p* = 0.1094). At one year after AHSCT, patients who progressed had significantly lower SCCSA at C1, C3 and C4 compared to those who did not (*p* = 0.044).

Patients who experienced disability progression after AHSCT showed a trend to lower C4 SCCSA at baseline compared to those who did not (median 36.8 mm^2^, range 36.6 – 37, and median 68.3 mm^2^, range 55.5 – 85.6, respectively; *p* = 0.1094; Fig. 2A). At 1 year following AHSCT, patients who progressed also showed a trend to lower SCCSA at C4 compared to those who did not progress (median 37.6 mm^2^ and 60.1 mm^2^, respectively; *p* = 0.0819, Fig. 2C).

At follow-up after AHSCT, a significant inverse correlation between SCCSA and EDSS was observed 1 year after transplant at vertebral body levels C1 (*r* = −0.78, *p* = 0.0081), C3 (*r* = −0.69, *p* = 0.0272), and C4 (*r* = −0.85, *p* = 0.0020). No correlations between SCCSA at follow-up and EDSS at follow-up were observed at any later time points. Correlation analysis between SCCSA and clinical-demographic characteristics is summarized in Table 2.

### 3.5. Brain atrophy

**Fig. 3.**
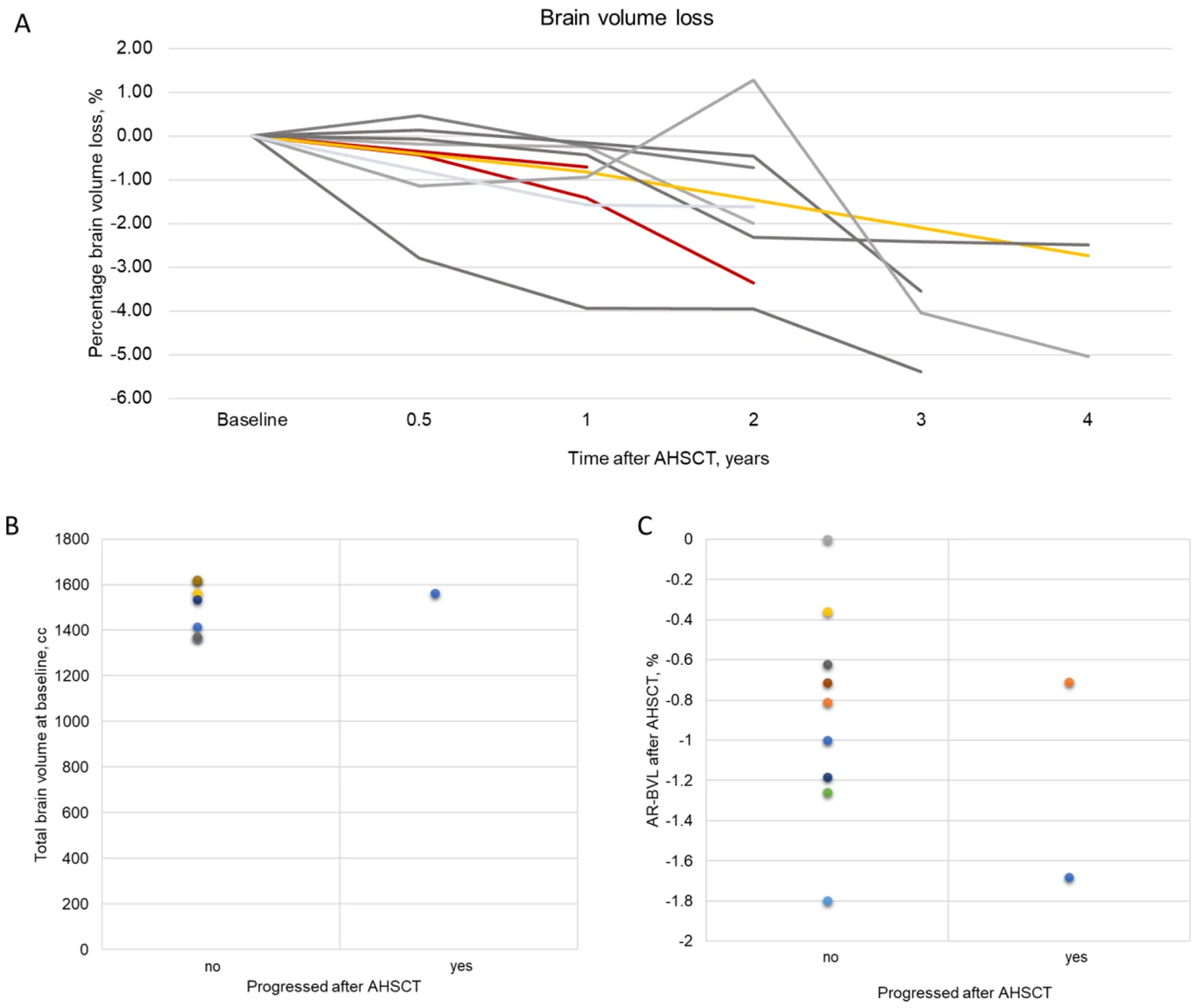
Brain volume loss after AHSCT (A). Patients who stabilized or improved are depicted in grey, whereas those who progressed or experienced a single-step EDSS increase are in red or yellow, respectively. Total brain volume at baseline (B) and AR-BVL (C) in cases who progressed and those who did not. No correlations between brain atrophy and disability outcomes were observed.

Brain atrophy change was analysed in 10 cases (3 SP-MS, 7 RR-MS; Fig. 3). The random coefficient model analysis (excluding a single outlier timepoint) showed that PBVC significantly decreased over time (*p* = 0.0027). No correlations were observed between AR-BVL and SCCSA or disability outcomes, except a weak positive correlation between AR-BVL and SCCSA percentage change at C2 level over the first year after AHSCT (*r* = 0.64; *p* = 0.0856) (data not shown).

### 3.6. Safety of AHSCT

No fatalities or life-threatening complications were observed in the patients included. Common adverse events following AHSCT were consistent with the literature (PA Muraro et al., 2017).

## 4. Discussion

In the present study, SCCSA was explored as a marker of disability outcomes in 11 MS patients treated with intermediate-intensity conditioning regimen AHSCT. Spinal cord atrophy, measured by SCCSA, is reported to better reflect disability as measured with EDSS than brain atrophy (Hidalgo de la Cruz et al., 2022), and it may be less susceptible to biological confounders than brain volume changes (Zivadinov et al., 2016).

Over a long follow-up of median 66 months, disability progression was observed in two patients, both affected by SP-MS at the time of AHSCT. The remaining SP-MS case experienced a single episode of EDSS increase, followed by prolonged stabilisation of disability, indicating that the progressive disease course was modified by the procedure. New focal inflammatory activity was suppressed in all the cases, and the safety profile of AHSCT was consistent with published data (Moore et al., 2019; Boffa et al., 2021).

Baseline SCCSA tended to be lower in SP-MS compared to RR-MS, as previously described (Lukas et al., 2015). The inverse correlation observed between age and spinal cord area was expected as a minor degree of atrophy is a feature of healthy ageing. However, in this patient population, baseline spinal cord volume may also be influenced by other factors, including MS severity and duration, and previous therapies. Accordingly, baseline SCCSA at C2 was inversely correlated with the number of previous DMTs, reflecting a more aggressive disease course correlating with lower spinal cord volume.

When exploring the role of SCCSA as a potential predictor of disability outcomes, a correlation between baseline SCCSA and EDSS change 1 to 2 years after AHSCT was observed, indicating that the cervical spinal cord area, particularly at the C4 level, may represent an important predictor of disability progression following AHSCT. However, as only three SP-MS patients were included in the study, and as disability progression was observed exclusively in two SP-MS cases, observation in larger patient populations is needed to exclude whether the correlation between lower SCCSA and progression was driven by the MS phenotype. Nonetheless, these data suggest that SCCSA may be an informative screening tool when assessing eligibility for AHSCT in MS patients with moderate disability, irrespective of their phenotypic classification. In fact, besides the well-known delay and difficulties in timely diagnosing the transition from RR- to SP-MS (Katz Sand et al., 2014), increasing evidence suggests that MS progresses along a continuum from relapsing to progressive disease (Vollmer et al., 2021). This is consistent with a recent study demonstrating that cervical spinal cord atrophy (i) was often present from the earliest disease stages, proceeding markedly faster in patients who later converted to SP-MS compared to those who did not, and (ii) predicted the speed of silent progression and conversion to progressive MS (Bischof et al., 2022). These data suggested that the latter phenomena are predominantly related to cervical cord atrophy, and that the diagnosis of SP-MS is a late recognition of neurodegenerative processes rather than a distinct disease phase (Bischof et al., 2022). These observations further support the opportunity for overcoming the current phenotypic classification by adopting a more comprehensive characterization of the different components of the disease process in each individual (Granziera et al., 2023); in this respect, spinal cord atrophy represents a promising “preclinical marker” of disability progression (Zeydan et al., 2022). Low SCCSA may represent a risk factor for disability progression after AHSCT with an additional mechanism. Assuming that low SCCSA indicates a low functional reserve, patients with low SCCSA may be at increased risk of disability progression once glial scarring (prompted by the rapid removal of inflammation induced by AHSCT) consolidates the axonal damage within pre-existing spinal cord lesions.

Previous longitudinal observations demonstrated that patients with MS who showed disability progression had a concomitant increase in the rate of cervical cord atrophy compared to those who were clinically stable, with most severe atrophy in the C4–5 region, corresponding to a portion of the cervical enlargement (Mina et al., 2021). In our study, longitudinal analyses did not show a significant decrease of SCCSA over time at the individual level. This suggests that AHSCT may slow down pathological rates of cervical spinal cord atrophy in MS, without inducing any early accelerations in spinal cord tissue loss; however, it cannot be excluded that the small sample size prevented us from finding significant longitudinal changes. At post-AHSCT timepoints, an inverse correlation was observed at year 1 between EDSS and SCCSA at vertebral body levels C1, C3, and C4, consistent with the literature indicating a strong correlation between cervical spinal cord volume (especially at C4-C5 levels) and EDSS in MS (Tsagkas et al., 2018; Mina et al., 2021; Seraji-Bozorgzad et al., 2015). No correlations between SCCSA and EDSS were observed at any later time points, possibly influenced by the small sample sizes at follow-up, as published literature suggested that the association between SCCSA and EDSS is preserved over time (Mina et al., 2021).

No correlations were observed between disability outcomes and baseline brain volume, nor with its longitudinal changes. A previous study using a busulfan-based regimen showed a trend for higher rates of brain atrophy in patients who progressed compared to those who did not, but baseline brain volume did not reliably predict disability outcomes after AHSCT (Lee et al., 2018). Several additional confounders, including lesion load, oedema due to recent inflammation, and pseudoatrophy/neurotoxicity of AHSCT may prevent the use of BVL as a biomarker in this setting. On the other hand, to our knowledge, no correlations between cervical spinal cord volume and biological confounders other than age are reported in the literature. Furthermore, lesion shrinkage and the resolution of inflammation in normal appearing white matter (pseudo-atrophy) likely contribute to a minor proportion of volume change in the spinal cord compared to the brain, plausibly due to different grey/white matter ratio of the two structures. Studies on healthy populations reported higher percentage volume loss in the brain than the spinal cord over time, with an average decrease of 0.27 % / year (De Stefano et al., 2016) and values ranging from 0 to 6 % over five decades (with high variability according to the spinal cord level and decade considered) (Kato et al., 2012), respectively. For these reasons, longitudinal monitoring of spinal cord atrophy could be useful, alone or in addition to brain atrophy, to monitor treatments effect, particularly in patients who are expected to show remarkable pseudoatrophy phenomena, such as those with aggressive forms of MS.

This study has several limitations. First, although this is a unique cohort of MS patients undergoing AHSCT, the sample size is small. However, AHSCT is a procedure reserved for highly selected patients, and 1446 transplants for MS were reported in the EBMT Registry up to July 2019 (Sharrack et al., 2019). The relatively small sample size does not allow generalization of these findings, that therefore should be considered as exploratory. Furthermore, it did not allow us to determine if AHSCT could affect the annual rate of cervical spinal cord atrophy, which is elevated in patients with MS compared to healthy controls, nor to perform sub-group analyses according to the MS phenotype (Cilingir and Akdeniz, 2022). Finally, subclinical disease activity in the spinal cord could not be excluded for all the patients, as spinal cord MRI was not routinely performed in cases treated before 2016. However, no active spinal cord lesions were observed in the two patients who progressed.

In conclusion, baseline SCCSA, but not its longitudinal changes or changes in BVL, predicted physical disability progression in the two years following AHSCT. Assessment of SCCSA may therefore provide valuable information when evaluating patients eligible for high-impact treatments such as AHSCT. In this setting, measurement of SCCSA may also aid the clinician in properly classifying the MS phenotype of the patient, due to the known challenges in timely recognizing transition to SP-MS. Further research is needed to confirm this exploratory finding in larger cohorts and provide generalizability of these results, and possibly identify a cut-off value that may be used as a predictor of treatment response.

## Figures and Tables

**Table 1:** Baseline clinical and demographic characteristics of the MS patients included.

	Total (*n* = 11)	RR-MS (*n* = 8)	SP-MS (*n* = 3)
Age at AHSCT, median years (range)	40 (24 – 48)	38 (24 – 44)	45 (27 – 48)
Disease duration, median years (range)	17 (5 – 24)	17.5 (5 – 24)	11 (7 – 23)
Treatment duration with DMTs, median years (range)	10 (4 – 22)	11 (4 – 22)	10 (4 – 17)
Previous DMTs, median number (range)	4 (1 – 6)	4 (1 – 5)	4 (4 – 6)
Relapses in the previous 2 years, median number (range)	2 (1 – 5)	2 (1 – 5)	1 (1 – 3)
EDSS, median (range)	2.5 (1.5 – 6.0)	2.25 (1.5 – 5.5)	6.0 (4.0 – 6.0)
Females, number (frequency)	10 (91 %)	7 (87 %)	3 (100 %)

Abbreviations: AHSCT, autologous haematopoietic stem cell transplantation; DMTs, disease-modifying treatment; EDSS, Expanded Disability Status Scale; RR-MS, relapsing-remitting multiple sclerosis; SP-MS, secondary-progressive multiple sclerosis.

**Table 2:** Significant Spearman correlations between spinal cord cross-sectional area (SCCSA) and clinical-demographic characteristics of the patients.

SCCSA level (timepoint)	Age at AHSCT, r (p value)	N prior DMTs, r (p value)	EDSS change at 1 y post-AHSCT, r (p value)	EDSS change at 2 y post-AHSCT, r (p value)	EDSS at 1 y post-AHSCT, r (p value)
C1 (baseline)	−0.68 (0.042)	n.s.	−0.71 (0.0496)	n.s.	n.s.
C2 (baseline)	n.s.	−0.68 (0.046)	n.s.	n.s.	n.s.
C3 (baseline)	n.s.	n.s.	−0.73 (0.0390)	n.s.	n.s.
C4 (baseline)	n.s.	n.s.	−0.89 (0.0073)	−0.90 (0.0374)	n.s.
C1 (1 y)	n.s.	n.s.	n.s.	n.s.	−0.78 (0.0081)
C3 (1 y)	n.s.	n.s.	n.s.	n.s.	−0.69 (0.0272)
C4 (1 y)	n.s.	n.s.	n.s.	n.s.	−0.85 (0.0020)

Abbreviations: AHSCT, autologous haematopoietic stem cell transplantation; C, cervical; DMTs, disease-modifying treatment; EDSS, Expanded Disability Status Scale; n.s., not-significant; SCCSA, spinal cord cross-sectional area; y, year.

## Data Availability

De-identified aggregated data will be shared upon written request to the corresponding author.
